# Study protocol of an open-label, single arm phase II trial investigating the efficacy, safety and quality of life of neoadjuvant chemotherapy with liposomal irinotecan combined with Oxaliplatin and 5-fluorouracil/Folinic acid followed by curative surgical resection in patients with hepatic Oligometastatic adenocarcinoma of the pancreas (HOLIPANC)

**DOI:** 10.1186/s12885-021-08966-3

**Published:** 2021-11-18

**Authors:** Florian Gebauer, Alexander Ioannis Damanakis, Felix Popp, Alexander Quaas, Fabian Kütting, Katrin Lutz, Swantje Held, Burkhard Deuß, Tobias Göser, Dirk Waldschmidt, Christiane Bruns

**Affiliations:** 1 Department of General, Visceral, Tumor and Transplantation Surgery, University of Cologne, Kerpener Strasse 62, 50937 Cologne, Germany; 2 Institute of Pathology, University of Cologne, Cologne, Germany; 3 Department of Gastroenterology, University of Cologne, Cologne, Germany; 4 ClinAssess GmbH, Leverkusen, Germany

**Keywords:** Chemotherapy, Clinical trials, Liver metastasis, Pancreatic cancer, Pancreatic surgery

## Abstract

**Background:**

According to current guidelines, treatment of patients with hepatic oligometastasis in pancreatic cancer is not reflected and systemic chemotherapy is recommended in those patients. Retrospective data suggest beneficial outcomes in patients with hepatic oligometastasis, though prospective data from clinical trials addressing this particular patient group is not available.

**Methods:**

In this single arm, phase-2 trial, survival data from patients receiving neoadjuvant chemotherapy followed by R0/R1 resection will be compared to historic data from patients with oligometastatic adenocarcinoma of the pancreas.

The clinical trial will focus on a well-defined patient collective with metastatic load limited to the liver as target organ with a maximum of five metastases. The combination of liposomal irinotecan (nal-IRI), oxaliplatin (OX) and 5-fluouracil (5-FU)/folinic acid (FA) (nal-IRI + OX+ 5-FU/FA, NAPOX) was chosen as neoadjuvant chemotherapy; the choice was based on an ongoing clinical study in which NAPOX appeared manageable, with promising anti-tumor activity in first-line treatment of patients with metastatic pancreatic adenocarcinoma.

In total 150 patients will be enrolled for this trial with an aim of 55 patients receiving a complete macroscopic synchronous tumor and metastatic resection.

**Discussion:**

This is the first clinical study to prospectively evaluate the value of multimodality therapy concepts in oligometastatic pancreatic cancer.

**Trial registration numbers:**

EudraCT 2019–002734-37; NCT04617457.

**Supplementary Information:**

The online version contains supplementary material available at 10.1186/s12885-021-08966-3.

## Background

In 2018, approximately 460,000 persons worldwide were diagnosed with pancreatic cancer and 430,000 patients died from this disease; the 5-year prevalence was 3.7% [1, 2]. Pancreatic cancer is among the deadliest malignancies and expected to become the second most common cause of cancer death by 2030 [3]. Once distant metastases have been detected, tumor or metastases resection is not recommended according to the current diagnostic and treatment guidelines, regardless of the location or number of metastases [4, 5]. Therefore, patients with hepatic oligometastatic adenocarcinoma of the pancreas currently receive palliative treatment.

In different tumor entities including colon and kidney cancer, synchronous tumor and hepatic metastases resection indicated survival benefits [6, 7]. Clinical studies are ongoing e.g. for gastric cancer (RENAISSANCE, NCT02578368) [8]. However, prospective clinical data on multimodal treatment in pancreatic cancer including complete tumor resection are not available so far.

Two recent retrospective studies showed that patients with hepatic oligometastatic pancreatic cancer benefited from complete tumor resection including resection of liver metastases: In an analysis of six European pancreas centers, 69 patients with pancreatic cancer were identified who had undergone synchronous tumor and hepatic metastases resection and who had a median OS of 14.5 months compared to 7.5 months in a control group without resection [9]. In another study, data of patients with oligometastatic pancreatic cancer undergoing tumor and metastases resection revealed a median OS of 12.3 months for patients with hepatic metastases [10]. Further reports including case reports revealed similar trends [11–16]. However, because of the limitations of retrospective study designs and of case reports as well as missing adequate control groups, the evidence level of these studies and reports is insufficient to allow for modifying diagnostic and treatment guidelines towards synchronous tumor and metastases resection in patients with oligometastatic pancreatic cancer.

The assumption that oligometastatic pancreatic cancer is biologically more similar to non-metastatic than to highly aggressive pancreatic cancer with multifocal tumor spread justifies the testing of a new therapeutic approach with curative intend for patients with a limited number of metastases [17, 18]. Additionally, retrospective data showed a survival benefit for patients that matched criteria of hepatic oligometastasis and only underwent standard palliative chemotherapy compared to patients with polymetastatic disease [19].

Since the establishment of novel, effective chemotherapy regimens, for the first time multimodal therapy in the post-gemcitabine monotherapy era is feasible to achieve long-term tumor control even in the metastatic stage [20–22]. novel chemotherapy regimes based on liposomal-irinotecan combined with 5-fluouracil and oxaliplatin showed promising anti tumor activity in metastasized pancreatic cancer in phase I/II trials [23]. Therefore, two approaches will be combined in this clinical trial: The goal is to test the efficacy and safety of neoadjuvant chemotherapy followed by complete synchronous resection of the primary tumor and hepatic metastases in curative intent in patients with adenocarcinoma of the pancreas and oligometastatic hepatic disease. The hypothesis of the clinical trial is that neoadjuvant chemotherapy sufficiently controls systemic disease and tumor progression, thus allowing the complete resection of the tumor and all hepatic metastases in case of stable disease or tumor response, so that these patients profit from it in terms of overall survival.

The study is planed as investigator initiated trial (IIT) and the sponsor of the study is the University of Cologne.

## Objectives and endpoints

### Objectives

The primary objective of the HOLIPANC trial is to assess the efficacy of neoadjuvant multimodal chemotherapy followed by complete tumor and metastases resection in patients with hepatic oligometastatic adenocarcinoma of the pancreas. Secondary objectives are to determine the efficacy and safety of the treatment concept and health-related quality of life (HR-QoL).

### Endpoints

The primary endpoint is overall survival after R0/R1 resection (OS-res) (only patients with R0/R1 resection). Secondary efficacy endpoints are OS of the entire patients cohort, R0/R1 resection rate and progression-free survival (PFS). Secondary safety endpoints are type, frequency and severity of adverse events with severity according to NCI CTCAE version 5.0 (neoadjuvant chemotherapy) and perioperative morbidity and mortality (Table 1).

**Table 1:** Objectives and endpoints of the HOLIPANC trial

1. **Objectives**	
1.1. **Primary Objectives**	
• To assess the efficacy of neoadjuvant multimodal chemotherapy followed by R0/R1 resection in patients with hepatic oligometastatic adenocarcinoma of the pancreas	
1.2. **Secondary Objectives**	
• To determine efficacy and safety of the treatment concept	
• To determine health-related quality of life (HR-QoL)	
1.3. **Other Exploratory Objectives**	
• To analyze HR-QoL-adjusted overall survival	
2. **Endpoints**	
2.1. **Primary Endpoint**	
• Overall survival after R0/R1 resection (OS-res) (only patients with R0/R1 resection)	
2.2. **Secondary Endpoints**	
**Efficacy**	
• R0/R1 resection rate after neoadjuvant chemotherapy	
• Overall survival	
• Progression-free survival (PFS) after R0/R1 resection according to RECIST v1.1	
**Safety**	
• Type, frequency and severity of adverse events with severity according to NCI CTCAE version 5.0 (neoadjuvant chemotherapy)	
• Perioperative morbidity and mortality	
**Health-Related Quality of Life**	
• HR-QoL according to EORTC QLQ-C30 and EORTC QLQ-PAN26 questionnaires	
2.3. **Other, Exploratory Endpoints**	
• HR-QoL-adjusted OS	

## Methods/study design

This is an interventional, open-label, non-randomised, multicentre, single-arm phase II clinical trial organized and sponsored by the University of Cologne. The study will be conducted at 10 centers in Germany.

**Fig. 1:**
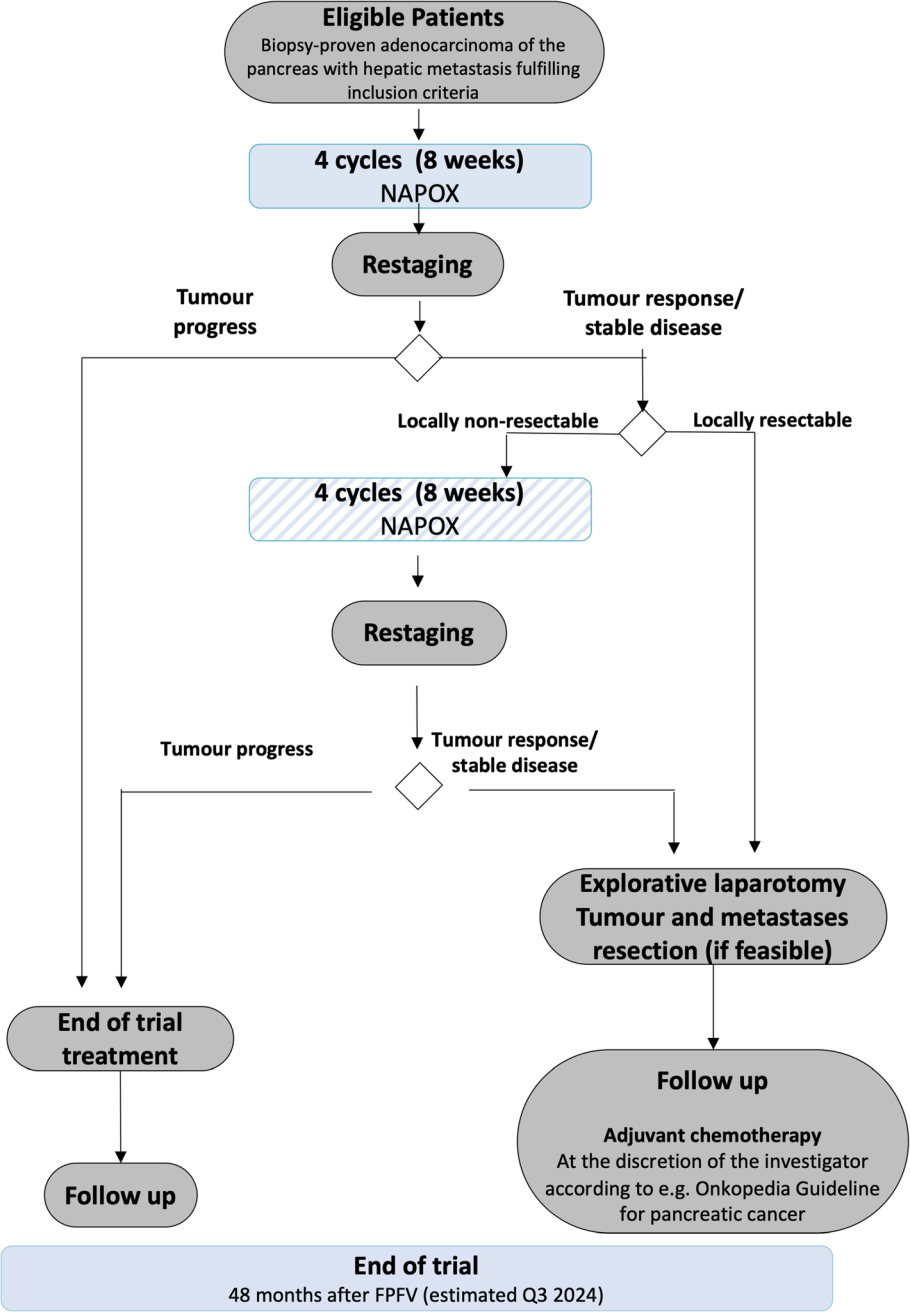
Flow chart of the entire study including biobanking project

Eligible patients with hepatic oligometastatic adenocarcinoma of the pancreas will receive neoadjuvant combination chemotherapy (liposomal irinotecan (nal-IRI), oxaliplatin (OX), 5-fluouracil (5-FU), folinic acid (FA) (NAPOX)) in cycles of 14 days (Fig. 1).

### Trial population

Patients with hepatic oligometastatic ductal adenocarcinoma of the pancreas fulfilling the inclusion criteria will be eligible to participate in this clinical trial.

### Inclusion criteria

1. Histologically confirmed diagnosis of treatment-naïve oligometastatic hepatic metastatic adenocarcinoma of the pancreas
  Definition of oligometastatic hepatic metastasis: 1 to 5 metastases in CT/MRI and/or contrast-enhanced ultrasound scan, which are potentially resectable or treatable by ablative procedures (Note 1: Patients also fulfill this inclusion criterion if a hepatic metastasis was partly or entirely removed as part of the diagnosis and is thus not detectable by CT/MRI and/or contrast-enhanced ultrasound scan at screening. Note 2: If more than 5 metastases are unexpectedly detected during surgery, it is not a violation of this inclusion criterion if the excess metastases had not been detectable by CT/MRI and/or contrast-enhanced ultrasound scan at screening.)
2. Measurable disease according to RECIST v1.1
3. ECOG performance status 0–1
4. Adequate renal, hepatic and bone marrow function, defined as

- Calculated creatinine clearance ≥60 mL/min according to CKD-EPI formula
- Total bilirubin ≤2 mg/dL; patients with biliary stent may be included if bilirubin level decreased to ≤2 mg/dL after stent insertion
- ALT and AST ≤5 × upper limit of normal (ULN)
- Absolute neutrophil count (ANC) ≥1.5 × 109/L
- Thrombocytes ≥100 × 10^9^/L
- Haemoglobin ≥9 g/dL
- aPTT ≤1.5 × ULN and Quick value ≥70%

1. Patients ≥18 years at the time of signing the informed consent
2. Females of childbearing potential (FCBPs) must agree to use highly effective contraceptive measures (Pearl index < 1) or practice true abstinence from any heterosexual intercourse for the duration of treatment and for at least 1 month after the last IMP administration (true abstinence is acceptable when this is in line with the preferred and usual lifestyle of the patient). A woman will be considered as being of childbearing potential unless she is at least 50 years old and moreover has gone through menopause for at least 2 years or has been surgically sterilized.
3. Males must agree to use condoms or practice true abstinence from any heterosexual intercourse for the duration of IMP treatment and at least 6 months after the last IMP administration (true abstinence is acceptable if this is in line with the patient’s preferred and usual lifestyle). Male patients must furthermore refrain from donating sperm during the clinical trial until at least 6 months after the last IMP administration.
4. Patient’s written informed consent prior to any trial-specific procedure
5. Patient’s legal capacity to consent to participation in the clinical trial

### Exclusion criteria

1. Acinar cell carcinoma and/or neuroendocrine carcinoma of the pancreas
2. Symptomatic clinically significant ascites
3. Evidence of any distant metastases other than oligometastatic hepatic metastases as defined in inclusion criterion 1.
4. Any tumor-specific pretreatment of the adenocarcinoma of the pancreas (including but not limited to surgery, radiation therapy, chemotherapy or ablative procedures)
5. Any malignancies other than adenocarcinoma of the pancreas in the 5 years before the start of the clinical trial except for adequately treated basal cell or squamous cell skin cancer, in situ cervical cancer, breast cancer, prostate cancer or superficial bladder tumors (Ta, Tis and T1)
6. Hypersensitivity to any of the IMPs or any of the excipients
7. Any major surgery within 4 weeks before the first IMP administration
8. Pregnant or breast-feeding female
9. Known chronic inflammatory bowel disease, bowel obstruction, chronic diarrhea, Grade ≥ 2 according to NCI CTCAE version 5.0
10. Peripheral polyneuropathy, Grade ≥ 2 according to NCI CTCAE version 5.0
11. Known interstitial lung disease or pulmonary fibrosis
12. Radiographic evidence of severe portal hypertension
13. Liver cirrhosis ≥ Child Pugh B
14. Cholestasis or cholangitis despite adequate biliary stenting; treatment with anti-infectious agents is permitted; patient must be disease-free and without anti-infectious treatment for 7 days before the first IMP administration
15. Active infection requiring systemic therapy
16. Known HIV seropositivity
17. Active or chronic Hepatitis B or Hepatitis C infection
18. Known glucoronidation deficiency (Gilbert’s syndrome) (specific screening not required)
19. Known complete dihydropyrimidine dehydrogenase (DPD) deficiency (specific screening according to the recommendations of the SmPC in effect for 5-FU; patients with a known complete DPD deficiency must be excluded; patients with a known partial DPD deficiency may be included at the discretion of the investigator)
20. Clinically significant cardiovascular or vascular disease or disorder ≤6 months before enrolment into the clinical trial (e.g., myocardial infarction, unstable angina pectoris, chronic heart failure NYHA ≥ Grade 2, uncontrolled arrhythmia, cerebral infarction)
21. Pulmonary embolism, deep venous thrombosis or arterial thromboembolism ≤6 months before the first IMP administration
22. Any other severe concomitant disease or disorder, which could influence patient’s ability to participate in the clinical trial and his/her safety during the trial or interfere with interpretation of results; e.g., severe hepatic, renal, pulmonary, cardiovascular, metabolic or psychiatric disorders
23. Requirement for live vaccination within 4 weeks before the first IMP administration and during neoadjuvant chemotherapy
24. Use of strong CYP3A4 inhibitors (Strong CYP3A4 inhibitors have to be discontinued at least one week prior to start of trial treatment.) Use of strong UGT1A1 inhibitors or strong CYP3A4 inducers unless there are no therapeutic alternatives
25. Treatment with nucleoside analogues such as brivudine within 4 weeks before the first IMP administration or requirement for concomitant antiviral treatment with brivudine or analogues
26. Participation in a clinical trial or experimental drug treatment within 4 weeks before the first IMP administration or within a period of 5 half lives of the substances administered in a clinical trial or during an experimental drug treatment before the first IMP administration, depending on which period is longest, or simultaneous participation in another clinical trial while taking part in this clinical trial until 28 days after last administration of any IMP
27. Continuing abuse of alcohol, drugs or medical drugs
28. Patient committed to an institution by virtue of an order issued either by the judicial or the administrative authorities
29. Patients possibly dependent from the investigator including the spouse, children and close relatives of any investigator

### Gender and age selection

Adults of all genders are eligible for this clinical trial.

### Tumor imaging

CT or MRI of abdomen with intravenous contrast agents according to specific protocols for imaging of the pancreas and local institutional practice; CT/MRI may be used for imaging of the abdomen, but investigators have to adhere to the same imaging method. Additive imaging such as MRI liver imaging with gadoxetate disodium (e.g., Primovist™) as contrast agent or, alternatively, a contrast-enhanced ultrasound scan will be required in case of unclear findings or suspicion of further hepatic metastases. Chest imaging by CT for detection of lung metastases. Additional imaging may be required in symptomatic patients if clinically indicated.

#### Treatment regimen

This is an interventional, open-label, non-randomised, multicentre, single-arm phase II clinical trial. Eligible patients with hepatic oligometastatic adenocarcinoma of the pancreas will receive neoadjuvant NAPOX chemotherapy in cycles of 14 days.

In patients with progressive disease during or after the first 4 cycles, neoadjuvant chemotherapy will be permanently discontinued (Table 2). Patients with tumor response or stable disease after the first 4 cycles according to RECIST v1.1 but a non-resectable primary tumor according to the evaluation of an interdisciplinary tumor board will receive 4 more cycles of neoadjuvant chemotherapy. Patients with tumor response or stable disease and a resectable primary tumor after the first 4 cycles will undergo explorative laparotomy and synchronous resection of the tumor and hepatic metastases, if feasible; these patients may receive 4 more cycles of neoadjuvant chemotherapy 2 4 weeks after the explorative laparotomy if the surgeon rated the primary tumor as non-resectable during the explorative laparotomy.

**Table 2:** Investigational medicinal products (IMP) used in the HOLPANC trial during neoadjuvant treatment

IMP	Dosing schedule [Day of 14-day cycle]	Dose [mg/m^**2**^]	Route of administration
Liposomal irinotecan (nal-IRI) anhydrous free base*	1	50	i.v. over about 90 min
Oxaliplatin (OX)	1	60	i.v. over 2 to 6 h
Folinic acid (FA)	1	400	i.v. over about 30 min
5-fluorouracil (5-FU)	1–2	2400	i.v. over about 46 h

* Equivalent to 60 mg/m^2^ irinotecan hydrochloride trihydrate

All patients who received a total of 8 cycles and who then have tumor response or stable disease according to RECIST v1.1 will undergo exploratory laparotomy surgery and synchronous resection of the tumor and hepatic metastases, if feasible according to the surgeon, 2 6 weeks after the last IMP treatment.

### Dose modifications and prerequisites for the start of a new cycle

Doses will be reduced for haematological and non-haematological toxicities. In case of concurrent toxicities, all dose modifications should be based on the worst preceding toxicity. Nal-IRI, OX and 5-FU doses may be reduced by two dose levels. Patients who require further dose reduction should discontinue treatment. The FA dose does not require adjustment, but FA has to be discontinued if 5-FU is permanently discontinued. Special attention should be paid to toxicities associated with 5-FU due to a deficiency in DPD activity. To this end, therapeutic drug monitoring may be considered. In general, the recommendations of the SmPC in effect for 5-FU have to be followed.

The following criteria for dosing delays and holidays due to adverse events apply:

- A cycle may be delayed, e.g., due to adverse events, by a maximum of 7 days.
- If adverse events would require a cycle delay of more than 7 days, this cycle is cancelled and the next cycle started according to schedule (e.g., Cycle 1 starts on Day 1; Cycle 2 is cancelled; Cycle 3 starts on Day 29).
- If adverse events require cycle delays of more than 28 days, treatment should be permanently discontinued. (e.g., Cycle 1 starts on Day 1; Cycle 2 and Cycle 3 are cancelled; Cycle 4 has to start on Day 43 or treatment will be discontinued).

### Adjuvant treatment

Adjuvant treatment will not be part of the trial treatment and may be given at the investigator’s discretion in accordance with the guidelines for pancreatic cancer [24].

### Surgical procedures

Exploratory laparotomy and resection should be performed within 2 to 6 weeks after the last IMP administration according to local institutional practice. All procedures necessary to prepare for surgery, during surgery and after surgery that are standard of care will also follow local institutional practice. Based on the intraoperative findings during exploratory laparotomy, the surgeon will evaluate and decide whether resection of the primary tumor in curative intent can be performed. Intraoperative rapid section analyses are obligatory for the pancreatic transection margin and optional for the resection margin of the common bile duct to ensure safe and margin-free resection. If the primary tumor is macroscopically non-resectable, intraoperative tumor biopsies are obligatory to confirm diagnosis of viable tumor cell at the origin of assumed non-resectability (e.g., superior mesenteric artery, common hepatic artery, celiac artery). For the removal of hepatic lesions, resection is recommended. However, radiofrequency ablation or microwave ablation of hepatic lesions are permitted on a case-by-case basis.

Resected tissue and tumor samples will be sent to and analyzed by the local pathology department.

#### Statistical considerations

This is an interventional, open-label, non-randomized, multi-center, single-arm phase II clinical trial. The primary objective is to assess the efficacy of neoadjuvant NAPOX chemotherapy followed by R0/R1 resection in patients with hepatic oligometastatic adenocarcinoma of the pancreas. To this end, overall survival after R0/R1 resection (OS-res) for patients with R0/R1 resection after neoadjuvant chemotherapy will be used as the primary endpoint.

Details of the statistical analysis will be described in a statistical analysis plan (SAP), which will be written before the data cut-off date for analysis. The statistical analysis will be carried out by ClinAssess GmbH, Leverkusen, the CRO contracted with this task by the sponsor.

### Statistical hypotheses and sample size determination

OS after R0/R1 resection (OS-res) will be used as the primary endpoint to assess the efficacy of NAPOX in patients with oligometastatic adenocarcinoma of the pancreas.

Hence, the hypotheses to be tested are:

- H_0_: median OS-res ≤10 months
- H_1_: median OS-res ≥14 months

The hypothesis will be tested with a one-sided log-rank test.

Since median OS-res of ≥14 months is expected, 53 patients and 42 OS-res events are required to test the null hypothesis with a power of 80% at a one-sided significance level of 0.1 (one-sample testing using log-rank test) if an accrual period of 14 months (period from FPI to resection approx. 4 months) and a minimum follow-up of 24 months after resection is assumed. The longer the follow-up duration, the higher is the power to detect a specific alternative effect size. Assuming a R0/R1 resection rate of 35%, 150 patients will be included in this clinical trial.

### Statistical analysis

All study practices and statistical methods are based on the ICH document ‘Statistical Principles for Clinical Trials’.

In general, standard descriptive methods will be used for all relevant data. Distribution parameters (mean, standard deviation, minimum, median and maximum) will be given for continuous data and counts and percentages for categorical data. For selected parameters, 95% confidence intervals will be presented. If required, appropriate tests (Fisher’s exact test or chi-square test for proportions, Wilcoxon rank-sum test for continuous parameters, log-rank test for time to event parameters, Cox proportional hazard model for hazard ratio) will be used for comparisons between groups. Multivariate analyses may be performed using appropriate regression models (e.g., Cox proportional hazard model, logistic regression). For tests and confidence intervals, a two-sided significance level α = 5% will be used except for the primary endpoint and unless otherwise specified. Missing data will not be replaced. Incomplete data will be imputed adequately if necessary.

Time-to-event data will be analyzed according to the Kaplan-Meier method (product-limit analysis). Patients who are not known to have had an event by the time of the analyses will be censored based on the last recorded date the patient was known to be event-free. HR-QoL data will be scored according to the algorithms described in the relevant scoring manuals. It is planned to evaluate the HR-QoL-adjusted OS, preferably using the Q-TWIST (quality-adjusted time without symptoms of disease progression of toxicity) method. Safety data will be continuously monitored, documented and reported as described in this protocol. The safety analysis includes type, incidence and severity of adverse events (severity according to CTCAE version 5.0), exposure to IMPs and laboratory parameters. If statistical methods described herein prove unsuitable during analysis, methods that are more appropriate will be used and any changes documented in the clinical study report (CSR).

#### Assessment of severity/intensity

For the grading of the severity/intensity of an adverse event (AE), the National Cancer Institute Common Toxicity Criteria for Adverse Events (NCI CTCAE) version 5.0 must be used. Medical and scientific judgment should be exercised in deciding whether expedited reporting is appropriate in other situations, such as important medical events that may not be immediately life-threatening or result in death or hospitalization but may jeopardize the patient or may require an intervention to prevent one of the outcomes listed in the definition above. These events should also usually be considered serious (SAE). All noxious and unintended responses to a medicinal product related to any dose should be considered adverse drug reactions (ADRs). The investigator must report the outcome of AEs and SAEs. All SAEs that have not resolved by the end of treatment visit or discontinuation of IMP treatment, whichever is later, must be followed until the outcome is recovered, recovered with squeal, unchanged/not recovered until death (death due to another cause) or death (due to the SAE). Investigators must report all SAEs immediately within 24 h of knowledge of the event.

#### Regulatory, ethical, legal and trial oversight considerations

The protocol was developed and approved by the sponsor. The clinical trial will be conducted in accordance with the ethical principles of the Declaration of Helsinki and ICH Good Clinical Practice guidelines and the applicable European and domestic law concerning the conduct of clinical studies. The local ethics committee of the University of Cologne (20–1544-AMG) and the local ethic committees of the participating centers throughout Germany approved the study and the protocol with the protocol number Uni-Koeln-4067 V6.0. The sponsor, the competent authorities and the ethics committee may stop the clinical trial or participation of a clinical trial site in the clinical trial for medical, safety, regulatory, administrative or other reasons consistent with ICH-GCP, the respective European Union’s and national legislation. The study is registered at the ‘European Union Drug Regulating Authorities Clinical Trials’ (EudraCT 2019–002734-37) and clinicalstrials.gov (NCT04617457).

### Informed consent

Informed consent is the free and voluntary agreement of a patient to participate in a clinical trial after having been informed of all aspects of the clinical trial relevant to the patient’s decision to participate. Patient’s written informed consent prior to any trial-specific procedure for study inclusion. The investigators must obtain freely given informed consent from every patient prior any procedures related to the clinical trial including the documentation of results of clinical routine procedures for trial purposes as set forth in the GCP ICH guidelines, the respective European Union’s and national legislation.

### Monitoring

Clinical site monitoring will be conducted to ensure that the rights and well-being of patients are protected, the reported trial data are accurate, complete and verifiable from source documents and that the conduct of the trial complies with the currently approved protocol/amendment(s), with GCP and with the applicable regulatory requirement(s). Periodic monitoring of the trial will be performed on-site in the trial centers, i.e., in terms of visits by Clinical Research Associates (CRAs), using a risk-based monitoring approach.

Following written Standard Operating Procedures (SOP), monitors will verify that the clinical trial is conducted and data generated, collected, recorded and reported according to GCP and the applicable regulatory requirements. The activities the CRA should carry out when relevant and necessary to the trial and the trial site will be described in detail in the monitoring plan. Among others, the activities will include the following:

•Availability of the patient’s informed consent
•Verification that the investigator is enrolling only eligible patients
•Verification that written informed consent was obtained before each patient’s participation in the trial
•Verifying that the investigator and site staff are adhering to the protocol and GCP
•Ensuring the completeness of the trial documents in the trial centre
•Source document verification by cross-checking the electronic CRFs against the investigator’s records
•Verifying that source documents and other trial records are accurate, complete, kept up-to-date and maintained
•Determination whether all AEs/SAEs are appropriately reported within the time periods required by GCP, the protocol, the sponsor and the applicable regulatory requirement(s)

### Audits

The sponsor may conduct or commission audits in the course of the trial, which are independent of and separate from routine monitoring or quality control functions, to evaluate trial conduct and compliance with the protocol, SOPs, GCP and the applicable regulatory requirements. The appointed auditors should be independent of the clinical trial and qualified by training and experience to conduct audits properly. The audit will be conducted according to an audit plan that is guided by the importance of the trial to submissions to regulatory authorities, the number of patients in the trial, the type and complexity of the trial, the level of risks to the trial patients and any identified problem(s).

### Trial oversight

Safety data will be assessed by a Data and Safety Monitoring Committee (DSMC) composed of individuals with relevant expertise, including surgery and oncology. Members of the DSMC should be independent from trial conduct and free of conflict of interest. The DSMC will operate under the rules of an approved DSMC charter.

### Confidentiality and data protection

The sponsor affirms the patient’s right to protection against invasion of privacy. All pertinent provisions of European and national data protection legislation in order to guarantee confidentiality and protection of privacy will be fully observed.

All records identifying the patients will be kept confidential and, to the extent permitted by the applicable laws and/or regulations, will not be made publicly available. The investigator must assure that the patient’s anonymity will be maintained and that the identities are protected from unauthorized parties. The investigator should maintain documents not for submission to the sponsor, e.g. patients’ written consent forms, in strict confidence. On the eCRFs and other documents patients should not be identified by their names or birth dates. All clinical and scientific data are collected under a patient-identification code.

All data transfer with the trial centres will be made without any exception via the patient-code. All participating trial centres are obliged to keep a strictly confidential patient identification list at a safe locked place.

Persons who are authorized by the sponsor or regulatory authorities (e.g. CRAs, auditors or representatives of regulatory authorities) may be permitted to patient-related data medical records relevant to the clinical trial for review or inspections respectively in accordance with local laws and the patient’s statement in the informed consent.

### Trial results and publication

The clinical trial will be registered in a public register in accordance with the recommendations of the International Committee of Medical Journal Editors’ (ICMJE).

A clinical trial report will be prepared within 1 yr after the end of the clinical trial. Within 1 yr after the end of the trial, the competent authority and the ethics committee will be supplied with the summary of the clinical trial report according to the regulatory requirements including the publication of results. The sponsor is furthermore required to post the results in the EudraCT database within 1 yr after the end of the trial.

### Translational research program

Residues of tumour and metastases tissue from biopsies and resection, as well as stool and blood samples collected before the start and during the clinical trial, will be used for translational research if the patient gives his/her consent to participating in the translational research programme. Eligible patients may participate in the clinical trial without consenting to translational research procedures.

Patients with hepatic oligometastatic adenocarcinoma of the pancreas usually do not undergo surgery; thus, biomaterials of these patients are not available. The main goals of this translational research programme therefore are to build a biobank with samples from this patient group and to perform comprehensive analyses signatures.

## Discussion

Once distant metastases have been detected tumor resection is not recommended neither according to German, nor to NCCN guidelines [4, 25]. In the palliative setting chemotherapy regimes such as FOLFIRINOX or gemcitabine/nab-Paclitaxel have been recently established, showing a significantly increased OS with a median of 11 and 8.5 months, respectively, compared to 7 or 6.7 months with gemcitabine mono therapy [20, 21]. Though, recommendations towards surgery in the metastasized stage are not given, based on individual decisions physicians have performed surgical resections in patients with hepatic metastases in the past. So far, prospective data facing this specific patient subgroup has not been published in any of the searched databases or registered as clinical trial (MEDLINE, the Cochrane library, clinicaltrials.gov, Deutsches Register Klinischer Studien (DRKS)). However, two recent studies showed for the first time data with a reasonable sample size of patients who underwent synchronous liver and primary tumor resection that patients with hepatic oligometastatic disease could potentially benefit from a complete tumor resection including resection of the liver metastases [9, 10]. These studies showed for the first time, apart from single case reports [11, 12, 15, 16, 26–28], a promising survival of 12.3 and 14 months OS, respectively. Due to several limitations in the retrospective study design and missing adequate control groups, the evidence level is by far too low to modify treatment guidelines towards synchronous liver resection in patients with oligometastatic PDAC.

The treatment of metastatic tumor diseases is a challenge not only in pancreatic cancer. More than 70% of gastrointestinal (GI) cancers are diagnosed with metastases either at the time of diagnosis (synchronous) or later (metachronous) [2, 29]. Interestingly, metastasis remains limited to a single lesion or few foci for longer periods of time in some patients, where local treatment suffices to obtain long term tumor control. However, current treatment of metastatic cancer is still based on the paradigm that metastatic spread beyond regional lymph nodes is considered uniformly as systemic disease. This concept results in non-selective, non-individualized systemic treatment without discriminating the substantial variety of clinical outcomes and potentially excluding patients accessible to curatively intended innovative local or multimodality treatments. Oligometastatic disease is poorly understood and its recognition is based upon imaging of metastatic lesions of limited number in distant organs. High-level evidence based on prospective randomized trials is lacking and clinical definitions of oligometastasis are still at a premature stage with very limited consensus across the scientific community [30]. So far, the single existing preliminary attempt of a definition of oligometastatic GI cancers is related only to colorectal cancer and has been formulated in the ESMO consensus guidelines [31]. Here, oligometastatic CRC is characterized by a limitation of the disease to few sites and lesions and multimodality treatment strategies including local therapies are recommended to improve disease control and clinical outcome in these patients. Distinct clinical courses of oligo- and polymetastasis occur in tumors of different origins and an inclusion of more than one entity is needed to identify common underlying mechanisms. In pancreatic cancer, the concept of oligometastasis has not been established in the clinical routine, moreover, today it is fully unknown if there is are real oligometastastic disease in pancreatic cancer compared to the stages we know from colorectal cancer. Based on a non-surgical patient collective, our group had proposed for the first time a definition for the oligometastatic disease in pancreatic cancer [19], however prospective data about clinical and molecular details of this particular patients group is still missing.

The HOLIPANC study aims therefore to evaluate the effectiveness of multimodal therapy in patients with the clinical picture of oligometastasis in pancreatic cancer. The combination of a highly effective polychemotherapy followed by a complete tumor resection will foster the intention of a possible curative treatment of these patients, a group of patients which has been considered as exclusively palliative until now. The primary objective of this single arm phase II study is to demonstrate the efficacy of this therapeutic concept in terms of overall survival. Depending on the results, further clinical study concepts will be developed to offer new individualized therapy options to selected patient groups in the future.

## Supplementary Information

**Additional file 1.** Table 1: Clinical trial schedule.

## Data Availability

The full study protocol and all regulatory documents can be provided by the sponsor upon request.

## Abbreviations

ADR: Adverse Drug Reaction
AE: Adverse event
CT: Computer tomography
CTCAE: Common Terminology Criteria for Adverse Events
CR: Complete response
DCR: Disease-control rate
DPD: Dihydropyrimidine dehydrogenase
DSMC: Data and Safety Monitoring Committee
eCRF: Electronic case report form
ECOG: Eastern Cooperative Oncology Group
FA: Folinic acid
FCBP: Females of childbearing potential
FPFV: First patient first visit
5-FU: 5-fluorouracil
GCP: Good clinical practice
GCP-V: GCP ordinance; German: *Verordnung über die Anwendung der Guten Klinischen Praxis bei der Durchführung von klinischen Prüfungen mit Arzneimitteln zur Anwendung am Menschen*
HR-QoL: Health-related quality of life
IB: Investigator’s brochure
ICH: International Conference on Harmonization
IEC: Independent ethics committee
ILD: Interstitial lung disease
IMP: Investigational medicinal product
ITT: Intention-to-treat
i.v.: Intravenous
MRI: Magnetic resonance imaging
MSI: Microsatellite instability
NCI CTCAE: National Cancer Institute Common Toxicity Criteria for Adverse Events
nal-IRI: Liposomal irinotecan
NAPOLI-1: Treatment regimen with liposomal irinotecan and 5-fluorouracil/folinic acid
NAPOX: Treatment regimen with liposomal irinotecan, oxaliplatin and 5-fluorouracil/folinic acid
NCI: National Cancer Institute
ORR: Objective response rate
OS: Overall survival
OX: Oxaliplatin
PFS: Progression-free survival
PR: Partial response
RECIST: Response evaluation criteria in solid tumours
RR: Response rate
SAE: Serious adverse event
SAR: Serious adverse reaction
SAS: Statistic software
SDV: Source data verification
SmPC: Summary of product characteristics
SUSAR: Suspected unexpected serious adverse reaction
TEAE: Treatment-emergent adverse event
TMF: Trial master file
TNM: Classification of malignant tumours

## References

[1] World Fact Sheet [https://gco.iarc.fr/today/data/factsheets/populations/900-world-fact-sheets.pdf].

[2] Siegel RL, Miller KD, Jemal A (2020). Cancer statistics, 2020. CA Cancer J Clin.

[3] Rahib L, Smith BD, Aizenberg R, Rosenzweig AB, Fleshman JM, Matrisian LM (2014). Projecting cancer incidence and deaths to 2030: the unexpected burden of thyroid, liver, and pancreas cancers in the United States. Cancer Res.

[4] Seufferlein T, Porzner M, Becker T, Budach V, Ceyhan G, Esposito I, Fietkau R, Follmann M, Friess H, Galle P et al: [S3-guideline exocrine pancreatic cancer]. Zeitschrift fur Gastroenterologie 2013, 51(12):1395–1440, DOI: 10.1055/s-0033-1356220. 10.1055/s-0033-1356220 24338757

[5] Tempero MA, Malafa MP, Chiorean EG, Czito B, Scaife C, Narang AK, Fountzilas C, Wolpin BM, Al-Hawary M, Asbun H (2019). Pancreatic Adenocarcinoma, Version 1.2019. J Nat Comprehen Cancer Network : JNCCN.

[6] Fahy BN, D'Angelica M, DeMatteo RP, Blumgart LH, Weiser MR, Ostrovnaya I, Gonen M, Jarnagin WR (2009). Synchronous hepatic metastases from colon cancer: changing treatment strategies and results of surgical intervention. Ann Surg Oncol.

[7] Martel G, Bertens KA, Canil C (2021). Surgical Management of Genitourinary Cancer Liver Metastases. Surg Oncol Clin N Am.

[8] Al-Batran S-E, Mueller DW, Vogel A, Winkler M, Lorenzen S, Novotny A, Pauligk C, Homann N, Jungbluth T, Goetze TO (2017). The RENAISSANCE (AIO-FLOT5) trial: effect of chemotherapy alone vs. chemotherapy followed by surgical resection on survival and quality of life in patients with limited-metastatic adenocarcinoma of the stomach or esophagogastric junction - a phase III trial of the German AIO/CAO-V/CAOGI. BMC Cancer.

[9] Tachezy M, Gebauer F, Janot M, Uhl W, Zerbi A, Montorsi M, Perinel J, Adham M, Dervenis C, Agalianos C, Malleo G, Maggino L, Stein A, Izbicki JR, Bockhorn M (2016). Synchronous resections of hepatic oligometastatic pancreatic cancer: disputing a principle in a time of safe pancreatic operations in a retrospective multicenter analysis. Surgery.

[10] Hackert T, Niesen W, Hinz U, Tjaden C, Strobel O, Ulrich A, Michalski CW, Buchler MW (2017). Radical surgery of oligometastatic pancreatic cancer. Eur J Surg Oncol.

[11] Frigerio I, Regi P, Giardino A, Scopelliti F, Girelli R, Bassi C, Gobbo S, Martini PT, Capelli P, D'Onofrio M (2017). Downstaging in stage IV pancreatic Cancer: a new population eligible for surgery?. Ann Surg Oncol.

[12] Iida T, Nakabayashi Y, Okui N, Shiba H, Otsuka M, Yanaga K (2014). Successful management of metachronous liver metastasis after pancreaticoduodectomy for pancreatic ductal carcinoma using hepatectomy and chemotherapy: a case report. Anticancer Res.

[13] Niess H, Conrad C, Kleespies A, Haas F, Bao Q, Jauch KW, Graeb C, Bruns CJ (2013). Surgery for metastasis to the pancreas: is it safe and effective?. J Surg Oncol.

[14] Singh A, Singh T, Chaudhary A (2010). Synchronous resection of solitary liver metastases with pancreaticoduodenectomy. JOP : J Pancreas.

[15] de Jong MC, Tsai S, Cameron JL, Wolfgang CL, Hirose K, van Vledder MG, Eckhauser F, Herman JM, Edil BH, Choti MA, Schulick RD, Pawlik TM (2010). Safety and efficacy of curative intent surgery for peri-ampullary liver metastasis. J Surg Oncol.

[16] Shrikhande SV, Kleeff J, Reiser C, Weitz J, Hinz U, Esposito I, Schmidt J, Friess H, Buchler MW (2007). Pancreatic resection for M1 pancreatic ductal adenocarcinoma. Ann Surg Oncol.

[17] Weichselbaum RR, Hellman S (2011). Oligometastases revisited. Nat Rev Clin Oncol.

[18] Zhao Y, Li J, Li D, Wang Z, Zhao J, Wu X, Sun Q, Lin PP, Plum P, Damanakis A, Gebauer F, Zhou M, Zhang Z, Schlösser H, Jauch KW, Nelson PJ, Bruns CJ (2019). Tumor biology and multidisciplinary strategies of oligometastasis in gastrointestinal cancers. Semin Cancer Biol.

[19] Damanakis AI, Ostertag L, Waldschmidt D, Kutting F, Quaas A, Plum P, Bruns CJ, Gebauer F, Popp F (2019). Proposal for a definition of "Oligometastatic disease in pancreatic cancer". BMC Cancer.

[20] Von Hoff DD, Ervin T, Arena FP, Chiorean EG, Infante J, Moore M, Seay T, Tjulandin SA, Ma WW, Saleh MN (2013). Increased survival in pancreatic cancer with nab-paclitaxel plus gemcitabine. N Engl J Med.

[21] Conroy T, Desseigne F, Ychou M, Bouche O, Guimbaud R, Becouarn Y, Adenis A, Raoul JL, Gourgou-Bourgade S, de la Fouchardiere C (2011). FOLFIRINOX versus gemcitabine for metastatic pancreatic cancer. N Engl J Med.

[22] Wang-Gillam A, Li CP, Bodoky G, Dean A, Shan YS, Jameson G, Macarulla T, Lee KH, Cunningham D, Blanc JF, Hubner RA, Chiu CF, Schwartsmann G, Siveke JT, Braiteh F, Moyo V, Belanger B, Dhindsa N, Bayever E, von Hoff DD, Chen LT, Adoo C, Anderson T, Asselah J, Azambuja A, Bampton C, Barrios CH, Bekaii-Saab T, Bohuslav M, Chang D, Chen JS, Chen YC, Choi HJ, Chung IJ, Chung V, Csoszi T, Cubillo A, DeMarco L, de Wit M, Dragovich T, Edenfield W, Fein LE, Franke F, Fuchs M, Gonzales-Cruz V, Gozza A, Fernando RH, Iaffaioli R, Jakesova J, Kahan Z, Karimi M, Kim JS, Korbenfeld E, Lang I, Lee FC, Lee KD, Lipton L, Ma WW, Mangel L, Mena R, Palmer D, Pant S, Park JO, Piacentini P, Pelzer U, Plazas JG, Prasad C, Rau KM, Raoul JL, Richards D, Ross P, Schlittler L, Smakal M, Stahalova V, Sternberg C, Seufferlein T, Tebbutt N, Vinholes JJ, Wadlow R, Wenczl M, Wong M (2016). Nanoliposomal irinotecan with fluorouracil and folinic acid in metastatic pancreatic cancer after previous gemcitabine-based therapy (NAPOLI-1): a global, randomised, open-label, phase 3 trial. Lancet.

[23] Wainberg Z, Boland P, Lieu C, Dayyani F, Macarulla T, Zhang B, Belanger B, Moore Y, Wang T, Maxwell F *et al*: A phase 1/2, open-label, dose-expansion study of liposomal irinotecan (nal-IRI) plus 5-fluorouracil/leucovorin (5-FU/LV) and oxaliplatin (OX) in patients with previously untreated metastatic pancreatic cancer. Ann Oncol 2019, 30 Suppl 4:iv123.

[24] Pancreatic Cancer [https://www.onkopedia.com/de/onkopedia/guidelines/pankreaskarzinom/].

[25] Tempero MA, Malafa MP, Behrman SW, Benson AB, Casper ES, Chiorean EG, Chung V, Cohen SJ, Czito B, Engebretson A (2014). Pancreatic adenocarcinoma, version 2.2014: featured updates to the NCCN guidelines. J Nat Comprehen Cancer Network : JNCCN.

[26] van Rijssen LB, Narwade P, van Huijgevoort NC, Tseng DS, van Santvoort HC, Molenaar IQ, van Laarhoven HW, van Eijck CH, Busch OR, Besselink MG*,* Dutch Pancreatic Cancer Group: Prognostic value of lymph node metastases detected during surgical exploration for pancreatic or periampullary cancer: a systematic review and meta-analysis. HPB : the official journal of the International Hepato Pancreato Biliary Association 2016, 18(7):559–566, DOI: 10.1016/j.hpb.2016.05.001. 10.1016/j.hpb.2016.05.001 PMC4925793 27346135

[27] Yamada S, Fujii T, Sugimoto H, Kanazumi N, Kasuya H, Nomoto S, Takeda S, Kodera Y, Nakao A (2009). Pancreatic cancer with distant metastases: a contraindication for radical surgery?. Hepato-Gastroenterol.

[28] Michalski CW, Erkan M, Huser N, Muller MW, Hartel M, Friess H, Kleeff J (2008). Resection of primary pancreatic cancer and liver metastasis: a systematic review. Dig Surg.

[29] Lambert AW, Pattabiraman DR, Weinberg RA (2017). Emerging biological principles of metastasis. Cell.

[30] Van Cutsem E, Cervantes A, Adam R, Sobrero A, Van Krieken JH, Aderka D, Aranda Aguilar E, Bardelli A, Benson A, Bodoky G (2016). ESMO consensus guidelines for the management of patients with metastatic colorectal cancer. Ann Oncol.

[31] Van Cutsem E, Cervantes A, Nordlinger B, Arnold D, Group EGW, Cervantes A, Pentheroudakis G, Felip E, Pavlidis N, Stahel RA et al: Metastatic colorectal cancer: ESMO Clinical Practice Guidelines for diagnosis, treatment and follow-up. Ann Oncol 2014, **25 Suppl 3**:iii1–9. 10.1093/annonc/mdu260 25190710

