# Supplementary material for: Study protocol of an open-label, single arm phase II trial investigating the efficacy, safety and quality of life of neoadjuvant chemotherapy with liposomal irinotecan combined with Oxaliplatin and 5-fluorouracil/Folinic acid followed by curative surgical resection in patients with hepatic Oligometastatic adenocarcinoma of the pancreas (HOLIPANC)
Source: BMC Cancer. 2021 Nov 18;21:1239. doi: 10.1186/s12885-021-08966-3 (PMC8600696; doi:10.1186/s12885-021-08966-3)
Supplement: Supplementary file 1 — Additional file 1. Table 1: Clinical trial schedule. [file 12885_2021_8966_MOESM1_ESM.docx]

| **Visit** | **Screening** | | **Treatment phase** | | | | | **End of treatment (EoT)/**  **pre-surgery** | | **Exploratory laparotomy/**  **resection ^24^** | **Post-surgery ^25^** | **Follow up** |
| --- | --- | --- | --- | --- | --- | --- | --- | --- | --- | --- | --- | --- |
|  |  |  | **Cycles**  **1‑4** | **Cycles**  **5‑8** | | **Tumour evaluation** | |  |  |  |  |  |
| **Visit Window** | Within 4 weeks before the first IMP administration  (unless otherwise specified) | | Day 1 of each 14-day cycle before the first IMP administration or within 48 hours before the first IMP administration (unless otherwise specified) | | | In the second week of Cycle 4 and in the second week of Cycle 8 (if applicable) | | 2‑6 weeks after the last IMP administration; before surgery  (if surgery will be performed) | | 2‑6 weeks after the last IMP administration | 4‑12 weeks after surgery (if surgery was performed); before the start of adjuvant therapy (if adjuvant therapy will be given) | About every 3 months (after EoT or post-surgery visit, whichever was the last visit) |
| Declaration of consent^1^ | × | |  |  | |  | |  | |  |  |  |
| Verification of in- and exclusion criteria | × | |  |  | |  | |  | |  |  |  |
| Medical history ^2^ | × | |  |  | |  | |  | |  |  |  |
| Physical examination ^3^ | × | | × | × | |  | | × | |  |  |  |
| Vital signs ^4^ | × | | × | × | |  | | × | |  |  |  |
| ECOG performance status | × | | × | × | |  | | × | |  | × |  |
| QoL questionnaires ^5^ |  | | ×^5^ | ×^5^ | |  | | × | |  | × |  |
| Haematology ^6^ | × | | × | × | |  | | × | |  |  |  |
| Clinical chemistry and coagulation ^7^ | × | | × | × | |  | | × | |  |  |  |
| HIV serology | × | |  |  | |  | |  | |  |  |  |
| HBV and HCV serology | × | |  |  | |  | |  | |  |  |  |
| Pregnancy test ^8^ | × | | × ^8^ | × ^8^ | |  | | × | |  |  |  |
| DPD deficiency test ^9^ | (×) | |  |  | |  | |  | |  |  |  |
| ECG ^10^ | × | |  | × ^10^ | |  | | × | |  |  |  |
| CA 19‑9 and CEA ^11^ | × | |  |  | | × | |  | |  | × |  |
| Tumour imaging ^12^ | × | |  |  | | (×)^12^ | | (×) ^12^ | |  | (×)^12^ |  |
| Tumour assessment ^13^ | × | |  |  | | × | | (×) | |  | × |  |
| Assessment of resectability status by an interdisciplinary tumour board ^14^ | × | |  |  | | × | |  | |  |  |  |
| Control/change of biliary stent ^15^ |  | | (×) ^15^ | (×) ^15^ | |  | |  | |  |  |  |
| Documentation of surgery ^16^ |  | |  |  | |  | |  | | × |  |  |
| Intraoperative evaluation of resectability ^17^ |  | |  |  | |  | |  | | × |  |  |
| Pathological assessment of resected tumour tissue ^18^ |  | |  |  | |  | |  | | × |  |  |
| Perioperative morbidity and mortality ^19^ |  | |  |  | |  | |  | | Continuously | |  |
| Adverse events and serious adverse events^20^ | Continuously | | | | | | | | |  |  |  |
| Major complications^26^ |  |  | | |  | |  | | Continuously^26^ | | | |
| Concomitant medication^21^ | Continuously | | | | | | | | |  |  |  |
| Documentation of progression (if applicable), tumour relapse (if successful R0/R1 resection), adjuvant chemotherapy, further palliative treatment and survival ^22^ |  | |  |  | |  | |  | |  | × | × |
| Biopsy sample for translational research (optional) ^23^ | × | |  |  | |  | |  | |  |  |  |
| Stool sample for translational research (optional) ^23^ | × | |  |  | |  | |  | |  |  |  |
| Blood samples for translational research (optional) ^23^ |  | | × ^23^ | × ^23^ | |  | | × | | × | × |  |
| Resection samples of tumour/metastases for translational research (optional) ^23^ |  | |  |  | |  | |  | | × |  |  |

1. Informed consent declaration has to be signed prior to any trial-specific examinations or procedures.
2. Medical history: including demography, relevant prior and ongoing concomitant diseases/disorders, medical history of the adenocarcinoma of the pancreas including date of diagnosis, TNM stage, histological confirmation, biliary stenting (yes/no) and kind of stent; number, size and location of hepatic metastases (unilobular/bilobular)
3. Physical examination: including measurement of weight and height (height only at screening)
4. Vital signs: blood pressure, pulse rate, body temperature
5. QoL questionnaires: questioning with EORTC QLQ‑C30 and EORTC QLQ‑PAN26 questionnaires on Day 1 of Cycles 1, 3, 5 (if applicable) and 7 (if applicable) before the first IMP administration or within 48 hours before the first IMP administration, at the end-of-treatment visit and at the post-surgery visit (if surgery was performed)
6. Haematology: complete blood count including leukocytes, lymphocytes, neutrophils, thrombocytes, haemoglobin, haematocrit
7. Clinical chemistry and coagulation: sodium, potassium, calcium, urea, serum creatinine, calculated creatinine clearance, lactate dehydrogenase (LDH), total protein, serum albumin, alkaline phosphatase, alanine aminotransferase (ALT), aspartate aminotransferase (AST), gamma-GT, total bilirubin, aPTT, Quick value
8. Pregnancy test: β-HCG in serum only in females of child-bearing potential (FCBP); within 7 days before the first IMP administration on Day 1 of Cycle 1, every 4 weeks during treatment and at the EoT/pre-surgery visit
9. Testing for DPD deficiency according to the recommendation of the SmPC in effect for 5-FU; no defined time frame for testing
10. ECG: at the screening visit, on Day 1 (±7 days) of Cycle 5 (if applicable), at the EoT/pre-surgery visit and at any time if clinically indicated
11. CA 19-9 and CEA: determination of serum levels within 4 weeks before the first IMP administration on Day 1 of Cycle 1, in the second week of Cycle 4, in the second week of Cycle 8 (if applicable) and post-surgery
12. Tumour imaging: CT or MRI of abdomen with intravenous contrast agents according to specific protocols for imaging of the pancreas and local institutional practice as standard clinical procedure according to medical guidelines at screening (1) within 4 weeks of the first IMP administration on Day 1 of Cycle 1, in the second week of Cycle 4, in the second week of Cycle 8 (if applicable) and post-surgery (documentation of post-surgery tumour imaging only required if post-surgery tumour imaging is performed at investigator’s discretion and as clinical standard of the trial center, not obligatory); CT/MRI may be used for imaging of the abdomen, but investigators have to adhere to the same imaging method during the clinical trial. Additive imaging such as MRI liver imaging with gadoxetate disodium (e.g., Primovist^TM^) as contrast agent or, alternatively, a contrast-enhanced ultrasound scan will be required in case of unclear findings or suspicion of further hepatic metastases at screening. Chest imaging by CT for detection of lung metastases to prevent non-indicated surgery as clinical standard during screening and in the second week of Cycle 8 (if applicable). Additional imaging may be required in symptomatic patients if clinically indicated. Intervals between CT/MRI should be at least 8 weeks; the interval between CT/MRI and surgery should not exceed 4 weeks. As screening procedure, respective tumour imaging (e.g. CT or MRI of abdomen, chest imaging by CT) performed outside the trial centre is permitted. **Note:** If a patient discontinues trial treatment prematurely, it is at the investigator’s discretion, after careful examination of the clinical findings, to decide whether further tumour imaging is clinically indicated to determine whether the patient is eligible for explorative laparotomy/resection. In this case, a further CT/MRI may be performed not earlier than 8 weeks after the previous CT/MRI. No further trial-related tumour imaging will be performed in the follow-up.
13. Tumour assessment according to RECIST v1.1: CT/MRI scans will be assessed locally at the trial site. Pseudonymised copies of the CT/MRI scans will be sent to the sponsor for an additional central radiological review of tumour response; the central review will not affect treatment decisions. **Note:** Any new lesion or unequivocal progression of any existing lesion irrespective of the RECIST v1.1 score qualifies as a criterion to discontinue IMP treatment.
14. Assessment of resectability status by an interdisciplinary tumour board: evaluation of resectability of the primary tumour based on imaging and recommendations in NCCN Clinical Practice Guidelines in Oncology for Pancreatic Adenocarcinoma, version 2.2017 (prior to surgery
15. Control/change of biliary stent: control of correct function of biliary stent by blood sample analysis (see clinical chemistry) within 48 hours before the first IMP administration of each new cycle; change of biliary stent in case of plastic stent every 8-10 weeks or if required according to clinical symptoms and/or signs of cholangitis; only for patients with biliary stents
16. Documentation of surgery: day of surgery, surgery technique, number and location of resected hepatic metastases and (if applicable) ablated hepatic metastases, type of ablation (if performed; radiofrequency ablation/microwave ablation with administered power and duration for each metastasis), duration of intensive care and in-patient hospitalization
17. Intraoperative evaluation of resectability: evaluation and decision by surgeon whether resection of the primary tumour in curative intent can be performed based on intraoperative findings during explorative laparotomy; intraoperative rapid section analyses are obligatory for the pancreatic transection margin and optional for the resection margin of the common bile duct to ensure safe and margin-free resection; if the primary tumour is macroscopically non-resectable, intraoperative tumour biopsies are obligatory to confirm diagnosis of viable tumour cells at the origin of assumed non-resectability (e.g., superior mesenteric artery, common hepatic artery, celiac artery).
18. Pathological assessment of resected tumour tissue: according to current guidelines for pathological tissue assessment and CRM-concept
19. Perioperative morbidity and mortality: continuous documentation for the period from surgery to 60 days after surgery: Dindo-Clavien score (Grad I‑V), postoperative pancreatic fistula (POPF; Grade BL, B, C), postpancreatectomy haemorrhage (PPH; Grade A‑C), delayed gastric emptying (DGE; Grade A-C), resurgery (yes/no), wound healing disorder (yes/no), other postoperative complications abscess, adhesion ileus, insufficiency biliodigestive anastomosis, insufficiency gastroenterostomy, abdominal wound dehiscence, other surgical complications, other non-surgical complications; for the period from surgery to 30 days after surgery: MTL30
20. Adverse events: Recording of type, frequency and severity of AEs (severity according to NCI CTCAE version 5.0) (continuously, starting with the day the informed consent was signed until 28 days after the last IMP administration. If the exploratory laparotomy/resection is performed before the end of the 28-day period after the last IMP administration, complications from the exploratory laparotomy/resection are not considered AEs and documented as perioperative morbidity and mortality unless the complications are considered related to trial treatment.)
21. Concomitant medication: Documentation of concomitant medication/treatment (continuously, starting with the day the informed consent was signed)
22. Documentation of progression (if applicable), tumour relapse (if successful R0/R1 resection), adjuvant chemotherapy, further palliative treatment and survival: With regard to tumour relapse after R0/R1 resection, only time and location of recurrence have to be documented in the eCRF.
23. Sampling of biomaterials for translational research (optional): according to the laboratory manual

Biopsy sample: residues of pretherapeutic formalin-fixed paraffin-embedded (FFPE) biopsies of the primary tumour and/or hepatic metastases

Stool sample: pretherapeutic stool sample

Blood samples: blood (3×10 mL EDTA tubes; 2×5 mL serum tubes) drawn on Day 1 of Cycles 1 and 5 before the first IMP administration or within 48 hours before the first IMP administration, at EoT/pre-surgery visit, on the day of surgery or within 48 hours before surgery and at the post-surgery visit

Resection samples of tumour/metastases: resection samples of the primary tumour (3×), each metastasis (3×, if feasible) and healthy control tissue (3×) after completion of routine histological and pathological measurements

1. Explorative laparotomy and surgery will be performed according to local institutional practice and in accordance with the Onkopedia guideline for pancreatic cancer (1). The listed assessments and procedures have to be performed as part of the clinical trial.
2. Adjuvant treatment will not be part of the trial treatment and may be given at the investigator’s discretion in accordance with the Onkopedia Guideline for pancreatic cancer (1). Additional assessment and procedures necessary before starting adjuvant therapy (only if adjuvant therapy will be given) do not have to be documented.
3. Major complications (regardless of causality assessment) occurring more than 28 days after the last administration of any IMP (including those during any subsequent adjuvant chemotherapy or palliative treatment lines of metastatic disease according to the treating physician’s decision that are not part of the study treatment) have to be documented continuously in the eCRF until death or end of clinical trial, whichever occurs first.
